# Supplementary material for: Systematic investigation of the link between enzyme catalysis and cold adaptation
Source: eLife. 2022 Jan 12;11:e72884. doi: 10.7554/eLife.72884 (PMC8754429; doi:10.7554/eLife.72884)
Supplement: Figure 3—source data 1. — a (Engqvist, 2018). b Alternatively reported to grow optimally at 65°C (Schröder et al., 1997). For consistency, curated values from Engqvist, 2018, are used in this work. [file elife-72884-fig3-data1.docx]

| **Variant** | **Organism** | **Isolation Origin** | **T_Growth_ (˚C)^a^** | **Reference** |
| --- | --- | --- | --- | --- |
| bbKSI | *Brevibacillus borstelensis* | Soil | 34 | Shida O, Takagi H, Kadowaki K, Komagata K. 1996. Proposal for Two New Genera, Brevibacillus gen. nov. and Aneurinibacillus gen. nov. *Int J Syst Bacteriol* **46**:939–946. doi:10.1099/00207713-46-4-939 |
| tKSI | *Comamonas testosteroni* | Soil | 29 | Marcus PI, Talalay P. 1956. Induction and purification of alpha- and beta-hydroxysteroid dehydrogenases. *J Biol Chem* **218**:661–674. |
| mbKSI | *Mycobacterium botniense* | Streamwater | 46 | Torkko P, Suomalainen S, Iivanainen E, Suutari M, Tortoli E, Paulin L, Katila ML. 2000. Mycobacterium xenopi and related organisms isolated from stream waters in Finland and description of Mycobacterium botniense sp. nov. *Int J Syst Evol Microbiol* **50**:283–289. doi:10.1099/00207713-50-1-283 |
| mhKSI | *Mycobacterium hassiacum* | Urine | 37 | Schröder KH, Naumann L, Kroppenstedt RM, Reischl U. 1997. Mycobacterium hassiacum sp. nov., a new rapidly growing thermophilic mycobacterium. *Int J Syst Bacteriol* **47**:86–91. doi:10.1099/00207713-47-1-86 |
| miKSI | *Mycobacterium mantenii* | Lymph node | 37 | van Soolingen D. 2009. Mycobacterium mantenii sp. nov., a pathogenic, slowly growing, scotochromogenic species. *Int J Syst Evol Microbiol* **59**:2782–2787. doi:10.1099/ijs.0.010405-0 |
| mmKSI | *Mycobacterium marinum* | Fish tubercles | 31 | Aronson J. 1926. Spontaneous Tuberculosis in Salt Water Fish. *J Infect Dis* **39**:315–320. |
| mpKSI | *Mycobacterium parmense* | Cervical lymph node | 37 | Fanti F, Tortoli E, Hall L, Roberts GD, Kroppenstedt RM, Dodi I, Conti S, Polonelli L, Chezzi C. 2004. Mycobacterium parmense sp. nov. *Int J Syst Evol Microbiol* **54**:1123–1127. doi:10.1099/ijs.0.02760-0 |
| msKSI | *Mycobacterium simiae* | Rhesus monkey | 37 | Karassova V, Weissfeiler J, Krasznay E. 1965. Occurrence of atypical mycobacteria in Macacus rhesus. *Acta Microbiol Acad Sci Hung* **12**:275–282. |
| mtKSI | *Mycobacterium thermoresistibile* | Soil | 39 | Tsukamura M. 1966. Adansonian Classification of Mycobacteria. *J Gen Microbiol* **45**:253–273. doi:10.1099/00221287-45-2-253 |
| naKSI | *Nocardia alba* | Soil | 29 | Li W-J, Jiang Y i., Kroppenstedt RM, Xu L-H, Jiang C-L. 2004. Nocardia alba sp.nov., a Novel Actinomycete Strain Isolated from Soil in China. *Syst Appl Microbiol* **27**:308–312. doi:10.1078/0723-2020-00270 |
| ntKSI | *Nocardia thailandica* | Pus | 29 | Kageyama A, Poonwan N, Yazawa K, Suzuki S, Kroppenstedt RM, Mikami Y. 2004. Nocardia vermiculata sp. nov. and Nocardia thailandica sp. nov. Isolated from Clinical Specimens. *Actinomycetologica* **18**:27–33. doi:10.3209/saj.18_27 |
| npKSI | *Nocardioides psychrotolerans* | Glacier | 20 | Liu Q, Xin Y, Liu H, Zhou Y, Wen Y. 2013. Nocardioides szechwanensis sp. nov. and Nocardioides psychrotolerans sp. nov., isolated from a glacier. *Int J Syst Evol Microbiol* **63**:129–133. doi:10.1099/ijs.0.038091-0 |
| oiKSI | *Oceanobacillus iheyensis* | Marine sediment | 29 | Lu J, Nogi Y, Takami H. 2001. Oceanobacillus iheyensis gen. nov., sp. nov., a deep-sea extremely halotolerant and alkaliphilic species isolated from a depth of 1050 m on the Iheya Ridge. *FEMS Microbiol Lett* **205**:291–297. doi:10.1111/j.1574-6968.2001.tb10963.x |
| psKSI | *Paenibacillus antarcticus* | Antarctic sediment | 15 | Montes MJ, Mercadé E, Bozal N, Guinea J. 2004. Paenibacillus antarcticus sp. nov., a novel psychrotolerant organism from the Antarctic environment. *Int J Syst Evol Microbiol* **54**:1521–1526. doi:10.1099/ijs.0.63078-0 |
| pgKSI | *Polaromonas glacialis* | Glacier | 20 | Margesin R, Spröer C, Zhang D-C, Busse H-J. 2012. Polaromonas glacialis sp. nov. and Polaromonas cryoconiti sp. nov., isolated from alpine glacier cryoconite. *Int J Syst Evol Microbiol* **62**:2662–2668. doi:10.1099/ijs.0.037556-0 |
| paKSI | *Pseudomonas aeruginosa* | Sliced boiled potatoes | 34 | Hugh R, Leifson E. 1964. The proposed Neotype Strains of Pseudomonas Aeruginosa (Schroeter 1872) Migula 1900. *Int Bull Bacteriol Nomencl Taxon* **14**:69–84. doi:10.1099/0096266X-14-2-69 |
| pKSI | *Pseudomonas putida* | Soil and water | 28 | Timmis KN. 2002. Pseudomonas putida: a cosmopolitan opportunist par excellence. *Environ Microbiol* **4**:779–781. doi:10.1046/j.1462-2920.2002.00365.x |
| rmKSI | *Rhodococcus marinonascens* | Marine sediment | 18 | Helmke E, Weyland H. 1984. Rhodococcus marinonascens sp. nov., an Actinomycete from the Sea. *Int J Syst Bacteriol* **34**:127–138. doi:10.1099/00207713-34-2-127 |
| ssKSI | *Shewanella halifaxensis* | Marine sediment | 16 | Zhao J-S, Manno D, Leggiadro C, O’Neil D, Hawari J. 2006. Shewanella halifaxensis sp. nov., a novel obligately respiratory and denitrifying psychrophile. *Int J Syst Evol Microbiol* **56**:205–212. doi:10.1099/ijs.0.63829-0 |
| spKSI | *Simplicispira psychrophilia* | Antarctic mosses | 20 | Terasaki Y. 1979. Transfer of Five Species and Two Subspecies of Spirillum to Other Genera (Aquaspirillum and Oceanospirillum) , with Emended Descriptions of the Species and Subspecies. *Int J Syst Bacteriol* **29**:130–144. |

**Figure 3—source data 1: KSI origins and organism growth temperatures.**

^a^ (Engqvist, 2018)

^b^ Alternatively reported to grow optimally at 65°C (Schröder et al., 1997). For consistency, curated values from (Engqvist, 2018) are used in this work.
