# Supplementary material for: Systematic investigation of the link between enzyme catalysis and cold adaptation
Source: eLife. 2022 Jan 12;11:e72884. doi: 10.7554/eLife.72884 (PMC8754429; doi:10.7554/eLife.72884)
Supplement: Figure 3—source data 2. — a (Engqvist, 2018). b Reported assay temperatures are the average of at least three measurements per experiment. c aAverage ± standard deviation from two to nine independent experiments with enzyme concentration varied by at least 5-fold. Values measured with substrate concentrations from 9 to 600 µM. Value of kcat/KM are less than 107 M–1 s–1 and thus unlikely to be limited by substrate binding. Reported assay temperatures are the average of at least three measurements per experiment. [file elife-72884-fig3-data2.pdf]

| Variant | $T_{Growth}$[a] | [E] | $T_{Assay}$ (°C)[b] | $k_{cat}$ ($s^{-1}$)[c] | $K_M$ (μM)[c] | $k_{cat}/K_M$ ($s^{-1}$ $M^{-1}$)[c] |
|---|---|---|---|---|---|---|
| bbKSI | 34°C | 25-500nM | 24.7 ± 0.5 | 1.7 ± 0.2 | 134 ± 54 | $(1.5 \pm 0.9) \times 10^4$ |
| tKSI | 29°C | 0.5-5nM | 26.0 ± 1.6 | 24.9 ± 12.2 | 40 ± 16 | $(6.1 \pm 1.1) \times 10^5$ |
| mbKSI | 46°C | 5-50nM | 25.1 ± 0.1 | 6.9 ± 0.6 | 40.5 ± 12 | $(1.8 \pm 0.4) \times 10^5$ |
| mhKSI | 37°C | 10-500nM | 25.2 ± 0.2 | 1.3 ± 0.1 | 92 ± 20 | $(1.5 \pm 0.2) \times 10^4$ |
| miKSI | 37°C | 5-100nM | 24.7 ± 0.8 | 1.6 ± 0.5 | 65 ± 37 | $(2.7 \pm 0.9) \times 10^4$ |
| mmKSI | 31°C | 0.25-5nM | 25.6 ± 1.3 | 106.3 ± 21.0 | 45 ± 21 | $(3.3 \pm 2.7) \times 10^6$ |
| mpKSI | 37°C | 10-50nM | 25.9 ± 1.4 | 3.4 ± 0.5 | 76 ± 28 | $(4.7 \pm 1.0) \times 10^4$ |
| msKSI | 37°C | 5-50nM | 25.0 ± 0.1 | 29.2 ± 7.7 | 80 ± 12 | $(3.7 \pm 1.1) \times 10^5$ |
| mtKSI | 39°C | 0.5-25nM | 26.0 ± 1.3 | 44.3 ± 7.7 | 47 ± 4 | $(9.6 \pm 2.1) \times 10^5$ |
| naKSI | 29°C | 10-50nM | 27.1± 0.6 | 15.9 ± 3.1 | 144 ± 52 | $(1.2 \pm 0.2) \times 10^5$ |
| ntKSI | 29°C | 10-100nM | 26.5 ± 1.2 | 4.8 ± 1.1 | 104 ± 49 | $(5.0 \pm 1.0) \times 10^4$ |
| npKSI | 20°C | 25-250nM | 24.8 ± 0.4 | 6.8 ± 2.0 | 368 ± 31 | $(1.8 \pm 0.4) \times 10^4$ |
| oiKSI | 29°C | 1-40nM | 25.9 ± 0.9 | 19.2 ± 7.6 | 107 ± 41 | $(2.6 \pm 1.4) \times 10^5$ |
| psKSI | 15°C | 50-500nM | 25.0 ± 0.0 | 1.7 ± 0.0 | 411 ± 30 | $(4.1 \pm 0.3) \times 10^3$ |
| pgKSI | 20°C | 0.5-25nM | 26.3 ± 0.9 | 18.1 ± 8.2 | 109 ± 86 | $(2.6 \pm 2.2) \times 10^5$ |
| paKSI | 34°C | 0.5-15nM | 26.8 ± 0.5 | 9.4 ± 2.3 | 21 ± 18 | $(7.7 \pm 5.9) \times 10^5$ |
| pKSI | 28°C | 1.5-15nM | 25.7 ± 1.1 | 11.2 ± 3.1 | 25 ± 13 | $(5.5 \pm 2.7) \times 10^5$ |
| rmKSI | 18°C | 5-50nM | 25.0 ± 0.0 | 10.3 ± 2.7 | 125 ± 13 | $(8.2 \pm 1.2) \times 10^4$ |
| ssKSI | 16°C | 5-50nM | 24.6 ± 0.6 | 8.7 ± 2.5 | 19 ± 3 | $(4.4 \pm 0.7) \times 10^5$ |
| spKSI | 20°C | 0.5-10nM | 26.4 ± 0.4 | 70.1 ± 21.6 | 77 ± 27 | $(9.8 \pm 3.4) \times 10^5$ |

**Figure 3—source data 2: Kinetic measurement of KSIs at 25˚C with substrate 5(10)-estrene-3,17-dione.**

[a] (Engqvist, 2018)

[b] Reported assay temperatures are the average of at least three measurements per experiment.

[c] average ± standard deviation from 2–9 independent experiments with enzyme concentration varied by at least 5-fold. Values measured with substrate concentrations from 9-600 μM. Value of $k_{cat}/K_M$ are less than $10^7$ $M^{-1}$ $s^{-1}$ and thus unlikely to be limited by substrate binding. Reported assay temperatures are the average of at least 3 measurements per experiment.
