# Supplementary material for: Systematic investigation of the link between enzyme catalysis and cold adaptation
Source: eLife. 2022 Jan 12;11:e72884. doi: 10.7554/eLife.72884 (PMC8754429; doi:10.7554/eLife.72884)
Supplement: Figure 3—source data 3. — a (Engqvist, 2018). b Reported assay temperatures are the average of at least three measurements per experiment. c Average ± standard deviation from two to four independent experiments with enzyme concentration varied by at least 2-fold. Values measured with substrate concentrations from 9 to 600 µM. [file elife-72884-fig3-data3.pdf]

Variant	T_{Growth}[a]	[E]	T_{Assay} (°C)[b]	k_{cat} (s^{-1})[c]	K_M (μM)[c]	k_{cat}/K_M (s^{-1} M^{-1})[c]
bbKSI	34°C	250-500nM	14.9 ± 0.2	0.27 ± 0.23	115 ± 77	(2.2 ± 0.6) × 10^3
tKSI	29°C	5-100nM	15.3 ± 0.6	4.9 ± 0.3	35 ± 1	(1.4 ± 0.1) × 10^5
mbKSI	46°C	50-100nM	15.3 ± 0.0	2.8 ± 0.2	52 ± 5	(5.3 ± 0.1) × 10^4
mhKSI	37°C	250-500nM	14.9 ± 0.2	0.6 ± 0.3	160 ± 102	(3.8 ± 0.9) × 10^3
miKSI	37°C	50-100nM	14.7 ± 0.1	0.42 ± 0.05	10.6 ± 0.9	(4.0 ± 0.8) × 10^4
mmKSI	31°C	2.5-5nM	15.0 ± 0.3	47.1 ± 7.6	21.2 ± 7.3	(2.5 ± 1.1) × 10^6
mpKSI	37°C	25-50nM	15.0 ± 0.0	1.16 ± 0.02	77 ± 30	(1.6 ± 0.6) × 10^4
msKSI	37°C	25-50nM	15.0 ± 0.1	8.2 ± 3.2	62 ± 39	(1.4 ± 0.3) × 10^5
mtKSI	39°C	5-15nM	15.2 ± 0.2	14.1 ± 1.6	53 ± 29	(3.2 ± 2.1) × 10^5
naKSI	29°C	25-50nM	14.9 ± 0.0	6.5 ± 1.0	116 ± 16	(5.6 ± 0.1) × 10^4
ntKSI	29°C	5-250nM	15.5 ± 0.6	1.9 ± 0.2	120 ± 21	(1.6 ± 0.1) × 10^4
npKSI	20°C	100-250nM	14.9 ± 0.2	3.2 ± 1.0	446 ± 116	(7.2 ± 0.4) × 10^3
oiKSI	29°C	1-50nM	15.3 ± 0.4	6.6 ± 0.4	73 ± 25	(9.9 ± 3.8) × 10^4
psKSI	15°C	250-500nM	15.3 ± 0.0	0.7 ± 0.1	500 ± 62	(1.5 ± 0.1) × 10^3
pgKSI	20°C	2.5-50nM	15.3 ± 0.8	6.8 ± 1.5	43 ± 9	(1.7 ± 0.7) × 10^5
paKSI	34°C	5-20nM	15.2 ± 0.3	1.3 ± 1.1	11 ± 5	(1.5 ± 1.5) × 10^5
pKSI	28°C	5-25nM	14.4 ± 1.0	4.9 ± 2.1	20 ± 13	(3.4 ± 2.1) × 10^5
rmKSI	18°C	25-50nM	15.4 ± 0.1	4.4 ± 0.8	147 ± 28	(3.0 ± 0.0) × 10^4
ssKSI	16°C	25-50nM	15.7 ± 0.2	2.5 ± 0.4	10 ± 1	(2.6 ± 0.2) × 10^5
spKSI	20°C	5-10nM	15.3 ± 0.3	20.2 ± 11.0	42 ± 8	(5.1 ± 3.7) × 10^5

Figure 3—source data 3: Kinetic measurement of KSIs at 15˚C with substrate 5(10)-estrene-3,17-dione.

[a] (Engqvist, 2018)

[b] Reported assay temperatures are the average of at least three measurements per experiment.

[c] average ± standard deviation from 2–4 independent experiments with enzyme concentration varied by at least 2-fold. Values measured with substrate concentrations from 9-600 μM.
