# Supplementary material for: Systematic investigation of the link between enzyme catalysis and cold adaptation
Source: eLife. 2022 Jan 12;11:e72884. doi: 10.7554/eLife.72884 (PMC8754429; doi:10.7554/eLife.72884)
Supplement: Supplementary file 1. [file elife-72884-supp1.docx]

| **Trait** | **Model** | **Highlighted experimental observations** | **References** |
| --- | --- | --- | --- |
| Flexibility (general) | Higher flexibility overcomes reduced motion at lower temperature, allowing enhanced catalysis. | Fish lactate dehydrogenase rate (*k*_cat_) correlates with average body temperature. Mollusk cytosolic malate dehydrogenase substrate affinity (*K*_M_) correlates with habitat temperature. Psychrophile α-amylase variant is faster than mesophilic and thermophilic variants. Chitobiase engineering stabilizes and reduces activity of psychrophilic enzyme. | (Feller and Gerday, 2003; Fields et al., 2015) |
| Flexibility (specific) | Surface flexibility decreases enthalpy and entropy activation terms, reducing temperature dependence of reaction. | Computational methods including molecular dynamics and empirical valence bond simulations of diverse enzyme systems, with specific focus on citrate synthase and trypsin variants, suggest importance of flexibility of surface residues in cold adaptation. | (Åqvist et al., 2017) |
| Heat capacity modulation | Altered temperature dependence of reaction reduces rate decrease as temperature is lowered | More negative Δ*C*_p_^‡^ has been observed in psychrophilic isopropylmalate dehydrogenase and α-glucosidase. In contrast, more negative Δ*C*_p_^‡^ has also been suggested as a driver of adaptation to higher temperature in reconstructed ancestral adenylate kinase sequences. | (Arcus et al., 2016; Nguyen et al., 2017) |
| Dynamic allostery | Partial unfolding arising from conformational entropy-enhancing mutation can affect *k*_cat_ and *K*_M_ | Dynamics-based regulation arising from mutations distal to the active site of mesophilic adenylate kinase affect substrate affinity and turnover, suggesting a mechanism of cold adaptation. | (Saavedra et al., 2018) |
