# Supplementary material for: Systematic investigation of the link between enzyme catalysis and cold adaptation
Source: eLife. 2022 Jan 12;11:e72884. doi: 10.7554/eLife.72884 (PMC8754429; doi:10.7554/eLife.72884)
Supplement: Supplementary file 2. [file elife-72884-supp2.docx]

| **Variant** | **Species** | **T_Growth_ (˚C)*** | **Pos. 16** | **Pos. 103** | **Pos. 40** | **Pos. 86** | **Pos. 120** | **Pos. 57** | **Pos. 32** | **Sequence** |
| --- | --- | --- | --- | --- | --- | --- | --- | --- | --- | --- |
| **pKSI** | *Pseudomonas putida* | 28 | Y | D | D | F | W | Y | Y | MNLPTAQEVQGLMARYIELVDVGDIEAIVQMYADDATVEDPFGQPPIHGREQIAAFYRQGLGGGKVRACLTGPVRASHNGCGAMPFRVEMVWNGQPCALDVIDVMRFDEHGRIQTMQAYWSEVNLSVREPQ |
| **tKSI** | *Comamonas testosteroni* | 29 | Y | D | D | F | F | Y | F | MNTPEHMTAVVQRYVAALNAGDLDGIVALFADDATVEDPVGSEPRSGTAAIREFYANSLKLPLAVELTQEVRAVANEAAFAFTVSFEYQGRKTVVAPIDHFRFNGAGKVVSMRALFGEKNIHAGA |
| **mhKSI** | *Mycobacterium hassiacum* | 37 | Y | S | D | W | W | Y | Y | MSTPQDNANTVHRYLEFVAKGQPDEIAALYADDATVEDPVGSEVHIGRQAIRGFYGNLENVQSRTEVKTLRALGHEVAFYWTLSIGGDEGGMTMDIISVMTFNDDGRIKSMKAYWTPENITQR |
| **mtKSI** | *Mycobacterium thermoresistibile* | 39 | Y | D | D | F | W | Y | Y | MTTVPDKTAAITDTVHRYLELVAQGRADEITELYADDATVEDPVGSDVHVGRQSIRKFYGNIENIKARTELLTLRVCGNEAAFLFRLEMDLGDNTMTIEPIDVMVFDADGRIASMKAYWN |
| **oiKSI** | *Oceanobacillus iheyensis* | 29 | Y | D | D | F | W | F | F | MPTEQEMKASLQKYLEGFNEGNSEKVISLFAEDARVEDPVGSEPLKGKASITTFFQQAIPSVKRLELAAPIRGSHGNAAAMAFNIYVEMEGKGAVIRCIDVMTFNDDGFIIDMKAYWGPEDVQS |
| **spKSI** | *Simplicispira psychrophilia* | 20 | Y | D | D | F | F | Y | Y | MPTPEHMQAAVRAYIAALNAGDIDAIVALYAEDATVEDPVGATPQRGLAEIRRFYSASLQMQLQVVLEGPVRAVANEAAFAFSVALVMDGQRLTIRPIDVMRFDDAGRITAMRAFFGPSNISHG |
| **pgKSI** | *Polaromonas glacialis* | 20 | Y | D | D | F | F | Y | Y | MPTPEHMQATVEAYVRALNASDLDAIVALYADDAVVEDPVGTAPKRGLAEIRAFYAGSLKLKLRVELEGQIRAVASEAAFAFSVSFEVKGQRTTIRPIDLFRFDDAGRIVQMRAFFGPANISAD |
| **paKSI** | *Pseudomonas aeruginosa* | 34 | Y | D | D | F | W | Y | Y | MISPQQVQEIMTRYVELVDACDIDGILALYARDALVEDPVGSPPHVGIEAVGRFYRNGLGRANARARRTGPVSASHAGSGAVPFCVDLEWNGRACSIQVIDVMEFDAGGLICSMKAYWGEANVVGRDAP |
| **bbKSI** | *Brevibacillus borstelensis* | 34 | Y | D | D | F | W | F | F | MNNSPMMKQALLAYVDAFNAGDAERLLALFAEEATVEDPVGLEPKRGRAEFEQFFRYAISGGAKLELVAPPRASFSNHAAVTFIVHTEMEGRAVGIHVTDVMTFDENGKIVHMRAFWGQDDVRTADSPNA |
| **msKSI** | *Mycobacterium simiae* | 37 | Y | S | D | W | W | Y | Y | MPSPEAITQTVNSYLTLLAKGATDEIVNLYTTDATIEDPIGADVLRGHDAVRAFYTAIQDAKKETELAEIRIGGNEAAFLWHLTLDAGDSRTRISPISVMTFDDQARVASMRAFWSPSDVRVL |
| **miKSI** | *Mycobacterium mantenii* | 37 | Y | S | D | W | W | Y | Y | MPSPEAITETVNRYLALVATGTADEIVTLYAADATIEDPIGSDIRRGHDAIRGFYAGFQDAKKDTELAELRISGSEAAFLWHLTLDAGDSRTRISPISTMSFDGDAKITSMRAFWSPADVQVL |
| **mmKSI** | *Mycobacterium marinum* | 31 | Y | E** | D | F | W | Y | Y | MPNSAERSQAITETVNRYMSVLADGDADDLVGFYADDATLEDPVGGEVHIGTRAIHGFYSAIAGLTRECELVSLRVCGNEAAFQFRLTVTSGDSKMRVEPIEVMVFDRSGKVAAMKAYWSAADVTHL |
| **mpKSI** | *Mycobacterium parmense* | 37 | Y | D | D | F | W | Y | Y | MRNAADRVQAITDTVNRYIELVAKGSADDLVELYADDATVEDPVGGEVHIGRQAIHGFYSAVDGVARECELVSLRVAGNEAAFLFRLTVTAGDHRMVIEPIDVMVFDDRGKVTAMKAYWSAANVTQG |
| **npKSI** | *Nocardioides psychrotolerans* | 20 | Y | E** | D | F | W | Y | Y | MVAPNADIRSTVQRYLDLVADGTSTEIVALYAPDATLEDPVGSEVLRGREAIGGFYAGLDGLAMTTNLVTLRVCAGHAAFHFEVVTDTGGMKFKMAPLEVMTFDGDGLITSMRAFWSDEDLVVDA |
| **naKSI** | *Nocardia alba* | 29 | Y | D | D | F | W | Y | Y | MASADDIRATVRKYVEAVGSGTAADVVALYREDATVEDPVGTEPHVGHAAITKFYENIEPLQRSTELFSVRVAGDSAAFSFRVVTTFGEQTFTLDPIDVMTFDEDARIVSMRAFWSQDDMVVG |
| **ntKSI** | *Nocardia thailandica* | 29 | Y | D | D | L** | W | Y | Y | MASPDDIRATVRRYVELVGTGTAADIAALYTEDATVEDPVGSAPHTGRAAIEKFYGALDGTTRHTELLTVRVAGDNAAFGLRVVTRAGDKTITIEPIDVMTFDADARITGMRAFWSASDIAFG |
| **mbKSI** | *Mycobacterium botniense* | 46 | Y | D | D | F | W | Y | Y | MRSAPERTQAITNAVHRYLGLLANGSVDDLVEMYAADATVEDPVGGEVHIGRQAIRSFYSALDGAERDCELVSLRVAGNEAAFQFRLTVATGGSVVRIEPIDVMAFADDGKVTAMKAYWSAADVTQLGSGDEAVRSGPGQSG |
| **ssKSI** | *Shewanella halifaxensis* | 16 | Y | D | D | F | W | Y | Y | MITEQFGLGVVSSYIEFLNNGNFEGIASLYSKNAIIEDPIGSDKIIGRTAIQDFYRQAVLGVHQVNQLGEVRVASNEIAFPFEVVLAKDPNLAISVIDIFKINAEGEIDSMRAFWGPGNVKSVSKPAPITA |
| **psKSI** | *Paenibacillus antarcticus* | 15 | Y | E** | D | I** | W | Y | F | MLEQPEIKQAMQQYIDHFNANDLESLLGLFSETASLEDPVGSIPIEGTEPIRQFYSKVVNGDTKIKLMTPICGSHSHSGAMAIEIETNAKGEKVVIQAIEIMSFDEFGKIMNLQVYWGKEDLNFS |
| **rmKSI** | *Rhodococcus marinonascens* | 18 | Y | D | D | F | W | Y | Y | MAPSAADIRKIVERYVAAVATGTADDVLSLYAEGATVEDPVGTEPRTSVDSLREFYSVLEPMKQTGELLTLRIAGNSAAFHFSLVTDLGEQKFEIAPIDVMTFDDDGKITSMKAYWGQDDMITRAD |
|  | (*) From Engqvist 2018 |  |  |  |  |  |  |  |  |  |
|  | (**) Novel active site feature not described in characterized KSIs previously (*P. putida*) numbering |  |  |  |  |  |  |  |  |  |
