# Supplementary material for: Systematic investigation of the link between enzyme catalysis and cold adaptation
Source: eLife. 2022 Jan 12;11:e72884. doi: 10.7554/eLife.72884 (PMC8754429; doi:10.7554/eLife.72884)
Supplement: Transparent reporting form [file elife-72884-transrepform1.docx]

***eLife’s* transparent reporting form**

We encourage authors to provide detailed information *within their submission* to facilitate the interpretation and replication of experiments. Authors can upload supporting documentation to indicate the use of appropriate reporting guidelines for health-related research (see [EQUATOR Network](http://www.equator-network.org/%20)), life science research (see the [BioSharing Information Resource](https://biosharing.org/)), or the [ARRIVE guidelines](http://www.plosbiology.org/article/info:doi/10.1371/journal.pbio.1000412) for reporting work involving animal research. Where applicable, authors should refer to any relevant reporting standards documents in this form.

If you have any questions, please consult our Journal Policies and/or contact us: [editorial@elifesciences.org](mailto:editorial@elifesciences.org).

**Sample-size estimation**

- You should state whether an appropriate sample size was computed when the study was being designed
- You should state the statistical method of sample size computation and any required assumptions
- If no explicit power analysis was used, you should describe how you decided what sample (replicate) size (number) to use

Please outline where this information can be found within the submission (e.g., sections or figure legends), or explain why this information doesn’t apply to your submission:

Sample sizes were not computed in advance.

**Replicates**

- You should report how often each experiment was performed
- You should include a definition of biological versus technical replication
- The data obtained should be provided and sufficient information should be provided to indicate the number of independent biological and/or technical replicates
- If you encountered any outliers, you should describe how these were handled
- Criteria for exclusion/inclusion of data should be clearly stated
- High-throughput sequence data should be uploaded before submission, with a private link for reviewers provided (these are available from both GEO and ArrayExpress)

Please outline where this information can be found within the submission (e.g., sections or figure legends), or explain why this information doesn’t apply to your submission:

The kinetic parameters *k*_cat_ and *K*_M_ (Figure 3C, 3D, Figure 3 Supplement 3, Figure 3 Source Data 2, Figure 3 Source Data 3) were determined by fitting the observed initial velocity of each enzyme variant reaction as a function of 5(10)EST concentration (9–600 μM; 6–7 different substrate concentrations per experiment) to the Michaelis–Menten equation. An independent experiment (“technical replicate”) is defined as an experiment carried out with a fresh dilution from concentrated enzyme stock. Reported values of *k*_cat_ and *K*_M_ are the average of 3–9 independent experiments with at least two different enzyme concentrations varied as specified in item legends. All data was included. A description of the enzyme preparation and measurements is provided in the Material and Methods section of the manuscript.

**Statistical reporting**

- Statistical analysis methods should be described and justified
- Raw data should be presented in figures whenever informative to do so (typically when N per group is less than 10)
- For each experiment, you should identify the statistical tests used, exact values of N, definitions of center, methods of multiple test correction, and dispersion and precision measures (e.g., mean, median, SD, SEM, confidence intervals; and, for the major substantive results, a measure of effect size (e.g., Pearson's r, Cohen's d)
- Report exact p-values wherever possible alongside the summary statistics and 95% confidence intervals. These should be reported for all key questions and not only when the p-value is less than 0.05.

Please outline where this information can be found within the submission (e.g., sections or figure legends), or explain why this information doesn’t apply to your submission:

For Figure 2 and Figure 2 Supplement 1,2, & 3, statistical analysis methods are described in the Materials and Methods. Exact values of n, measure of center (median), and 95% confidence intervals of the median calculated by bootstrapping are reported in figure legends or on figures themselves. All data can be found in Figure Source Data Files, as well as the analysis used may be found in included python and RStudio scripts. For Figure 2 Figure Supplement 3 the slope and the significance of the slope are reported on figure panels and summarized in Figure 2E & 2F.

For Figure 3C & Figure 3 Supplement 3A, the bar graphs show mean values of technical replicates and the error bars represent standard deviations, as described above for enzyme measurements above. This information is also represented on the scatter plots of Figure 3D & Figure 3 Supplement 3B, 3C, 3D.

For Figure 4, exact values of n and the coefficient of determination R^2^ are represented on the figure panels. Median and confidence intervals is specified in the figure legend.

P-values, regardless of significance, are reported in Figure 2 and Figure 2 supplements, including source data files.

(For large datasets, or papers with a very large number of statistical tests, you may upload a single table file with tests, Ns, etc., with reference to sections in the manuscript.)

**Group allocation**

- Indicate how samples were allocated into experimental groups (in the case of clinical studies, please specify allocation to treatment method); if randomization was used, please also state if restricted randomization was applied
- Indicate if masking was used during group allocation, data collection and/or data analysis

Please outline where this information can be found within the submission (e.g., sections or figure legends), or explain why this information doesn’t apply to your submission:

For Figure 2D, Figure 2 Supplement 1, Figure 2 Supplement 2, we derived a control distribution of enzyme rates by comparing enzyme variant rates originating from different organisms with identical TGrowth values. We calculated the rate ratio and its reciprocal (*k*_max_/*k*_min_ and *k*_min_/*k*_max_) for each reaction, as described in the Materials and Methods.

**Additional data files (“source data”)**

- We encourage you to upload relevant additional data files, such as numerical data that are represented as a graph in a figure, or as a summary table
- Where provided, these should be in the most useful format, and they can be uploaded as “Source data” files linked to a main figure or table
- Include model definition files including the full list of parameters used
- Include code used for data analysis (e.g., R, MatLab)
- Avoid stating that data files are “available upon request”

Please indicate the figures or tables for which source data files have been provided:

Source Data is available for Figure 1, Figure 2, Figure 3, and Figure 4. Every effort was made to be transparent about data and analysis, and relevant short scripts for analysis are also included for Figure 2.
